# CK2-dependent phosphorylation of occludin regulates the interaction with ZO-proteins and tight junction integrity

**DOI:** 10.1186/1478-811X-11-40

**Published:** 2013-06-10

**Authors:** Max J Dörfel, Julie K Westphal, Christian Bellmann, Susanne M Krug, Jimmi Cording, Sonnhild Mittag, Rudolf Tauber, Michael Fromm, Ingolf E Blasig, Otmar Huber

**Affiliations:** 1 Institute of Biochemistry II, Jena University Hospital, Friedrich-Schiller-University Jena, Nonnenplan 2, 07743 Jena, Germany; 2 Institute of Laboratory Medicine and Pathobiochemistry, Charité – Universitätsmedizin Berlin, Hindenburgdamm 30, 12200 Berlin, Germany; 3 Leibniz-Institute of Molecular Pharmacology, Robert-Rössle-Str. 10, 13125 Berlin, Germany; 4 Institute of Clinical Physiology, Charité – Universitätsmedizin Berlin, Hindenburgdamm 30, 12200 Berlin, Germany; 5 Current addresses: MJD, Cold Spring Harbor Laboratory, Stanley Institute for Cognitive Genomics, 500 Sunnyside Blvd, Woodbury, NY 11797 USA; 6 JKW, Oxacell AG, Helene-Lange-Str. 12, 14469 Potsdam, Germany

## Abstract

**Background:**

Casein kinase 2 (CK2) is a ubiquitously expressed Ser/Thr kinase with multiple functions in the regulation of cell proliferation and transformation. In targeting adherens and tight junctions (TJs), CK2 modulates the strength and dynamics of epithelial cell-cell contacts. Occludin previously was identified as a substrate of CK2, however the functional consequences of CK2-dependent occludin phosphorylation on TJ function were unknown.

**Results:**

Here, we present evidence that phosphorylation of a Thr400-XXX-Thr404-XXX-Ser408 motif in the C-terminal cytoplasmic tail of human occludin regulates assembly/disassembly and barrier properties of TJs. In contrast to wildtype and T400A/T404A/S408A-mutated occludin, a phospho-mimetic Occ-T400E/T404E/S408E construct was impaired in binding to ZO-2. Interestingly, pre-phosphorylation of a GST-Occ C-terminal domain fusion protein attenuated binding to ZO-2, whereas, binding to ZO-1 was not affected. Moreover, Occ-T400E/T404E/S408E showed delayed reassembly into TJs in Ca^2+^-switch experiments. Stable expression of Occ-T400E/T404E/S408E in MDCK C11 cells augments barrier properties in enhancing paracellular resistance in two-path impedance spectroscopy, whereas expression of wildtype and Occ-T400A/T404A/S408A did not affect transepithelial resistance.

**Conclusions:**

These results suggest an important role of CK2 in epithelial tight junction regulation. The occludin sequence motif at amino acids 400–408 apparently represents a hotspot for Ser/Thr-kinase phosphorylation and depending on the residue(s) which are phosphorylated it differentially modulates the functional properties of the TJ.

## Background

Tight junctions (TJs) represent the most apical cell-cell contacts in epithelial and endothelial tissues and play a central role in the maintenance of tissue integrity. In forming multiple anastomosing strands surrounding the cells they allow close contacts between opposing cytoplasma membranes which form a barrier regulating the passage of small molecules, ions, water and pathogens, thereby protecting subepithelial and -endothelial tissues from the external environment [1-3]. In separating apical and basolateral membrane compartments, TJs contribute to the maintenance of cell polarity. In addition to these more structural functions, TJs act as highly dynamic signaling platforms, which integrate numerous signaling pathways and regulate a variety of cellular processes involved in differentiation, proliferation and apoptosis [4-6].

As an integral part of TJs, a set of transmembrane proteins including claudins and the tight junction-associated MARVEL protein (TAMP) family members occludin, tricellulin and MarvelD3 define TJ structure and function. The extracellular loops of these four-transmembrane proteins form homophilic or heterophilic trans-interactions with TJ transmembrane proteins on opposing cell surfaces thereby sealing the intercellular space and determining the permeability characteristics of epithelial cell layers [5,7,8]. On the other hand the intracellular N- and C-terminal domains of these transmembrane proteins assemble TJ-associated proteins such as zonula occludens (ZO) proteins ZO-1, -2 and -3, 7H6, cingulin and symplekin which are essential for the association of TJs with the actin cytoskeleton and for assembly and maintenance of TJs [9]. Interestingly, some of these proteins reveal the typical dual function of Nacos (nuclear and adhesion complexes) proteins affecting adhesive activity and nuclear gene transcription [10]. Moreover, many TJ proteins are targets of protein kinases, which modulate assembly, stability and functional properties of TJs [4].

When occludin was identified as the first integral TJ protein it was recognized as a central component common to epithelial and endothelial TJs [11] which is able to form TJ-like strands [12]. The initial finding that occludin knockout mice showed fully developed TJs in epithelial tissues [12] with no major defects in barrier properties indicated that occludin has no essential barrier function. In contrast, more detailed analysis of the complex phenotypes observed in these knockout animals suggested that occludin may play a role in epithelial differentiation and proliferation [13]. Knockdown of occludin in Madin Darby canine kidney (MDCK) II cells resulted in altered composition of claudin proteins thus affecting permeability characteristics [14]. Meanwhile, there is a significant body of evidence indicating that occludin is important for the regulation of TJ structure and integrity and that this function is critically regulated by phosphorylation events [15,16]. Different factors and stimuli such as cytokines [17], vascular endothelial growth factor (VEGF) [18], redox changes [19], oxidized phospholipids [20], bile acids [21] lysophosphatic acid or phorbol ester [16] have been identified that alter phosphorylation of occludin on serine, threonine or tyrosine residues thereby affecting TJ properties. Several kinases including c-Yes [22], c-Src [23], protein kinase C (PKC) [24,25], phosphatidylinosite-3-kinase (PI3K) [26] as well as protein phosphatases like the receptor tyrosine phosphatase DEP-1 [27] or the protein phosphatases PP2A and PP1A [28] have been reported to interact with or regulate the phosphorylation of occludin.

The casein kinases CK1 and CK2 represent ubiquitously expressed serine/threonine kinases common to eukaryotic organisms. Both kinases target the C-terminal domain of occludin, whereas CK1 in addition is able to phosphorylate the occludin N-terminal domain [29-33]. However, the functional consequences of CK1- or CK2-dependent phosphorylation on TJs are not completely clear to date.

Here, we focused on the role of CK2 regulating TJ function. CK2 is a constitutive active master kinase involved in the regulation of multiple cellular processes including cell proliferation, apoptosis, gene expression and of the circadian rhythm [34]. Its subcellular localization appears to define its specific targets in response to different signals [34,35]. CK2 activity is frequently upregulated in cancer and contributes to the regulation of signaling pathways such as Wnt and NFκB signaling [36,37]. CK2 is composed of two regulatory β-subunits and two enzymatically active α-subunits (α, α´), which phosphorylate the consensus sequence motif S/T-X_1-2_-E/D and can use both ATP and GTP as phosphate donors.

Previous studies have identified Thr375 and Ser379 in *Xenopus laevis* occludin [30] and amino acids Thr403 and Ser407 in mouse occludin [33] as CK2 phosphorylation sites. Recently, we identified Thr400 as a third CK2 phosphorylation site in human occludin in addition to Thr404 and Ser408 which correspond to the sites identified in Xenopus and mouse occludin [31]. In the current study, phospho-site mutations were used to investigate the role of CK2-dependent phosphorylation of occludin on TJ function. Occ-T400E/T404E/S408E which mimics constitutively phosphorylated occludin showed reduced binding to ZO-2. Moreover, MDCK C11 cells stably transfected with this occludin construct revealed increased paracellular resistance without changes in transcellular barrier properties. In addition, Occ-T400E/T404E/S408E induces enhanced disassembly and delayed reassembly of TJs in calcium-switch experiments. In contrast, an Occ-T400A/T404A/S408A mutation neither influences barrier properties nor affects assembly and localization of occludin in calcium-switch experiments compared to wildtype occludin.

## Results

### Occludin directly interacts with CK2

**Figure 1:**
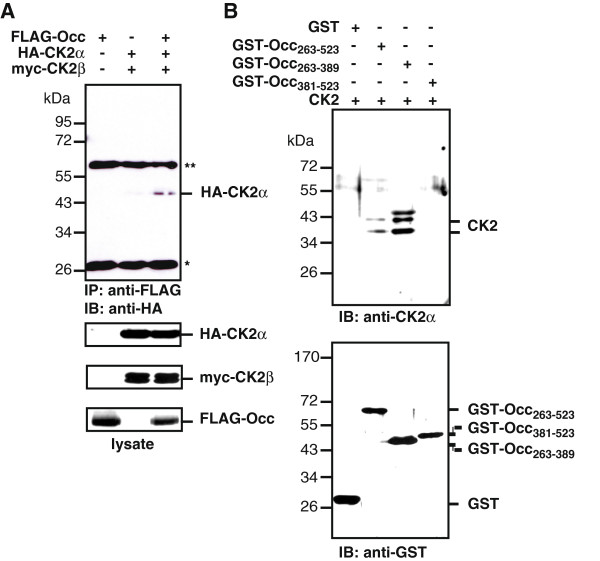
**CK2 interacts with occludin. A**) HEK-293 cells were transiently transfected with FLAG-occludin and HA-CK2α and myc-CK2β as indicated, and after 24 h cells were lysed. Co-immunoprecipitations were performed with anti-FLAG M2 antibody and protein complexes were analyzed by SDS-PAGE and Western blotting with anti-HA antibody. Lysate controls are shown. **B**) Purified recombinant GST-fusion proteins of the occludin C-terminal cytoplasmic domain and deletion constructs thereof were incubated with recombinant CK2 protein and protein complexes were pulled down with GSH-agarose beads to show a direct interaction. After analysis by SDS-PAGE and Western blotting binding of CK2 was detected with an anti-CK2α antibody. Equal amounts of GST-fusion proteins were pulled down with GSH beads as shown in the lower panel. ** indicates heavy chain, and * indicates light chain of the precipitating antibody. The presented figures are representatives of at least three independent experiments.

Previous *in vitro* studies have shown that the occludin C-terminal domain is phosphorylated by CK2 [30,31]. To test whether an interaction of CK2 with occludin also occurs in cells, co-immunoprecipitation experiments were performed. HEK-293 cells were transiently transfected with full-length FLAG-occludin (FLAG-Occ) and/or HA-CK2α and myc-CK2β, and subsequently FLAG-Occ was precipitated with anti-FLAG M2 antibody. In cells transfected with both occludin and CK2, common complexes containing FLAG-Occ and CK2α were pulled down (Figure 1A). In control experiments where FLAG-Occ or CK2 were transfected alone, no binding was detectable. Pull-down assays with purified recombinant GST-OccC fusion proteins containing the C-terminal cytoplasmic tail of human occludin (amino acids 263–523) and CK2 further confirmed these data. GST-fusion proteins of the C-terminal cytoplasmic domain of occludin (GST-OccC) or a deletion construct of this domain (GST-Occ_263-389_) containing the membrane proximal part of the cytoplasmic domain, both bind to CK2. In contrast, the distal part of the C-terminal cytoplasmic domain (GST-Occ_381-523_) revealed no affinity to CK2 (Figure 1B).

### Phosphomimetic mutation of the CK2-phosphorylation sites in occludin attenuates interaction with ZO proteins

**Figure 2:**
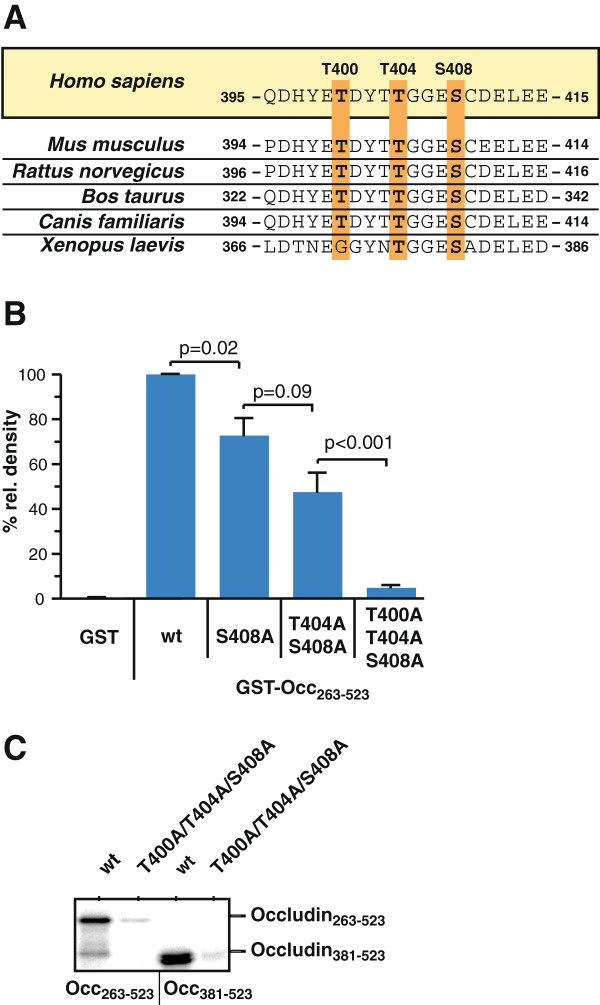
**CK2 phosphorylates a highly conserved T400/T404/S408 motif in the cytoplasmic tail of occludin. A**) Alignment of amino acid sequences containing the CK2 phosphorylation motif in occludin from different species. Conserved threonine (T) and serine (S) residues targeted by CK2 are highlighted. **B**) Densitometric analysis of *in vitro* phosphorylation experiments using purified recombinant GST-fusion proteins of the occludin C-terminal domain and the indicated Ser/Thr to Ala mutated constructs. Data represent mean values +/− SEM of 4 independent experiments. **C**) Full-length cytoplasmic tail (GST-Occ263-523) and the C-terminal half of it (GST-Occ381-523) and the corresponding triple alanine mutated proteins were *in vitro* phosphorylated with recombinant CK2 and subsequently analyzed by autoradiography. The triple alanine mutations abrogated phosphorylation by CK2. The presented figure is a representative of 3 independent experiments.

Recently we have shown that CK2 phosphorylates human occludin in a cluster of amino acids including residues Thr400, Thr404 and Ser408 [31]. Sequence alignment of occludin from different species demonstrates that Thr400, Thr404 and Ser408 are located in a highly conserved region (Figure 2A). Successive mutation of these amino acids to alanine gradually reduces the CK2-dependent phosphorylation of occludin. Simultaneous mutation of all three amino acids to alanine completely abolishes the CK2-dependent phosphorylation (Figure 2B and C).

**Figure 3:**
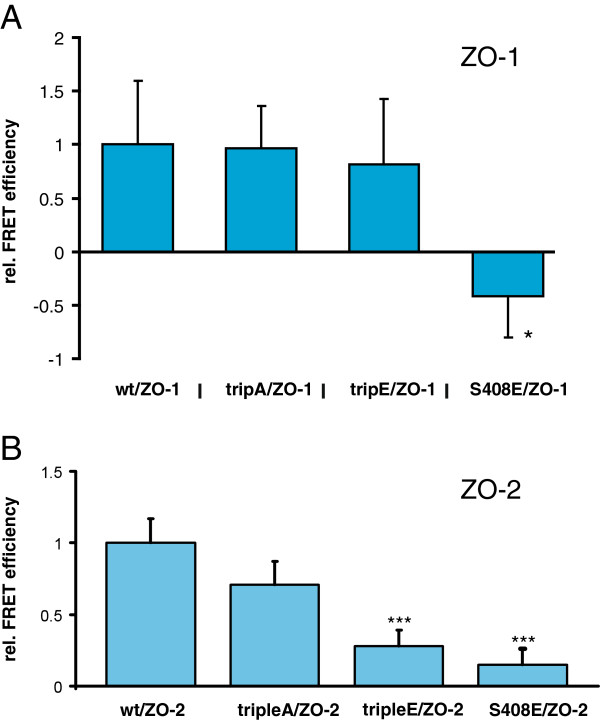
**Phosphorylation of T400/T404/S408 differentially affects binding to ZO-1 and ZO-2. A**) The indicated occludin (Occ) constructs were transiently cotransfected into HEK-293 cells. The phosphomimetic Occ S408E shows reduced cis-interaction along the cell membrane with ZO-1 protein (S408/ZO-1, n = 12) compared to Occ wt with ZO-1 (wt/ZO-1, n = 26), p < 0.05 (*), Mann–Whitney test, one-tailed. The Occ triple E mutant (TripE/ZO-1, n = 30) as well as the triple A mutant (TripA/ZO-1; n = 34) were without effect. **B**) In contrast to ZO-1, phosphomimetic triple E and S408E occludin constructs exhibited reduced cis-interaction with the ZO-2 protein (tripleE/ZO-2, n = 37; S408/wt, n = 42) compared to Occ wt (wt/ZO-2, n = 35), p < 0.011 (***), Mann–Whitney test, one-tailed. The Occ mutant triple A (TripA/ZO-2; n = 32) was without significant effect. Cis-interaction was measured as fluorescence resonance energy transfer (FRET) efficiency using a FRET assay at cell-cell contacts between two cotransfected cells. The Occ constructs were N-terminal fusions with yellow fluorescent protein (YFP) and that of ZO-1 and ZO-2 were C-terminally fused with cyan fluorescent protein (CFP).

Previous studies have demonstrated that tyrosine phosphorylation of the occludin C-terminal cytoplasmic domain impairs its interaction with ZO-proteins [23,38]. Otherwise, mutagenesis of Thr403 and Thr404 to alanine targeting a PKCη site was reported to reduce junctional localization of ZO-1 [39]. In this context, we next wanted to address if CK2-dependent phosphorylation also affects binding of occludin to ZO-proteins. When we pulled down ZO-1 with GST-OccC T400E/T404E/S408E or GST-OccC T400A/T404A/S408A from MDCK C11 lysates we did not see a significant difference in binding of endogenous ZO-1 compared to the wild-type construct (not shown). This observation was discrepant to data reporting that CK2-dependent phosphorylation of occludin on S408 affects binding to ZO-1 [40]. Therefore we decided to analyze ZO-1 binding by FRET in HEK293 cells, which were cotransfected with different occludin and ZO-1 constructs. Consistent with our biochemical assays, the triple-mutant occludin constructs again did not differ from wildtype occludin in binding to ZO-1. However, an occludin S408E mutant showed reduced binding to ZO-1 as reported previously [40] (Figure 3A) indicating a differential effect of the single compared to the triple-phosphorylation. Interestingly, further FRET analyses performed to analyze ZO-2 binding to the mutated occludin constructs revealed, that occludin-T400E/T404E/S408E as well as occludin-S408E are both impaired in binding to ZO-2 (Figure 3B).

**Figure 4:**
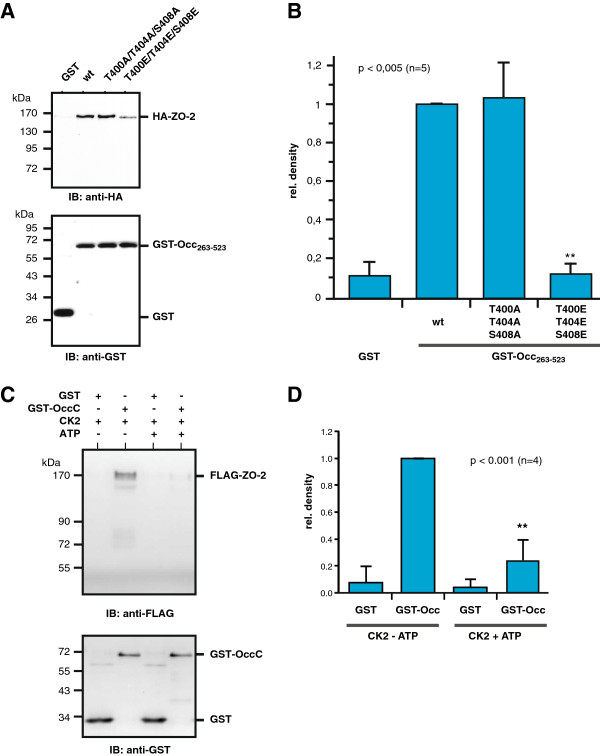
**Impaired binding of ZO-2 to the phospho-mimetic Occ-T400E/T404E/S408E construct. A**) The indicated GST-Occ cytoplasmic tail fusion proteins were used to pull down HA-ZO-2 from transiently transfected MDCK C11 cells with GSH-agarose beads. Isolated protein complexes were analyzed by Western blotting with anti-HA antibody. Equal amounts of GST- fusion proteins were pulled down as detected with an anti-GST antibody in the lower panel. **B**) Quantification of 5 independent experiments as shown in (A). **C**) Purified GST-occludin C-terminal domain (GST-OccC) was prephosphorylated *in vitro* by purified CK2 and subsequently used to pull down FLAG-tagged ZO-2 from transiently transfected HEK-293 cells. Association of FLAG-ZO-2 was analyzed by Western blotting with the anti-FLAG M2 antibody. **D**) Densitometric quantification of 4 experiments as shown in (**C**).

To further confirm this, pull-down experiments with purified GST-OccC or corresponding phospho-site mutated proteins were performed with cell lysates of MDCK C11 cells transfected with HA-tagged ZO-2. Wildtype and the triple phospho-deficient T400A/T404A/S408A mutant of GST-OccC strongly interacted with HA-ZO-2, whereas binding to the phospho-mimetic GST-OccC T400E/T404E/S408E construct was significantly reduced (Figure 4A and B). Co-immunoprecipitation assays corroborated this finding. Endogenous ZO-2 from MDCK C11 cells transiently transfected with full-length FLAG-tagged occludin variants formed a complex with wildtype Occ-FLAG_3_ and Occ-FLAG_3_ T400A/T404A/S408A. Association of Occ-FLAG_3_ T400E/T404E/S408E with ZO-2 was again reduced (Additional file 1: Figure S1). Finally, when GST-OccC was prephosphorylated *in vitro* by purified recombinant CK2 and used in pull-down experiments with lysates from FLAG-ZO-2-transfected HEK-293 cells, association of FLAG-ZO-2 was significantly reduced (Figure 4C and D). Together with FRET experiments, these results indicate, that phosphorylation of occludin by CK2 abrogates occludin interaction with ZO-2. This suggests that phosphorylation of all three CK2 sites in occludin differentially affects binding of occludin to ZO-1 or ZO-2 and in addition induces a different effect compared to phosphorylation of S408 alone.

### CK2-dependent phosphorylation of occludin does not affect its localization to tight junctions

**Figure 5:**
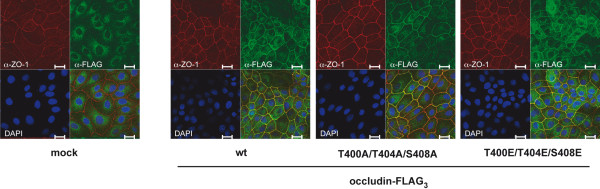
**Localization of wildtype occludin-FLAG**_**3 **_**and the corresponding T400/T404/S408 mutated constructs.** MDCK C11 cells stably transfected with the indicated FLAG_3_-tagged occludin constructs were stained with anti-ZO-1 (red) and anti-FLAG M2 (green) antibodies and nuclei were stained with DAPI (clones: mock 3.1, wt 1.1, T400A/T404A/S408A 3.1, T400E/T404E/S408E 4.1). Representative images of the indicated clones are shown. Images were taken on a confocal laser-scanning microscope. The lower right panel represents a merged image of the other three images. Bar, 20 μM.

To study the physiological effects induced by the CK2-site mutant occludin proteins, MDCK C11 cells were stably transfected with FLAG-tagged occludin constructs, and two clones of each vector-transfected (mock), Occ-FLAG_3_, Occ-FLAG_3_-T400A/T404A/S408A- and Occ-FLAG_3_-T400E/T404E/S408E-transfected cells, respectively, were selected for further analyses. In a first step, the localization of the occludin constructs was examined by confocal immunofluorescence microscopy after staining with the anti-FLAG M2 antibody. All three Occ-FLAG_3_ variants were detectable in the plasma membrane and colocalized with ZO-1 (Figure 5) and ZO-2 (Additional file 2: Figure S2) in TJs. Comparable results were obtained with occludin constructs carrying an N-terminal FLAG_3_-tag (not shown). In consequence, we conclude that neither the phosphorylation-deficient nor the phospho-mimetic occludin variants are affected in their transport and localization to TJs.

**Figure 6:**
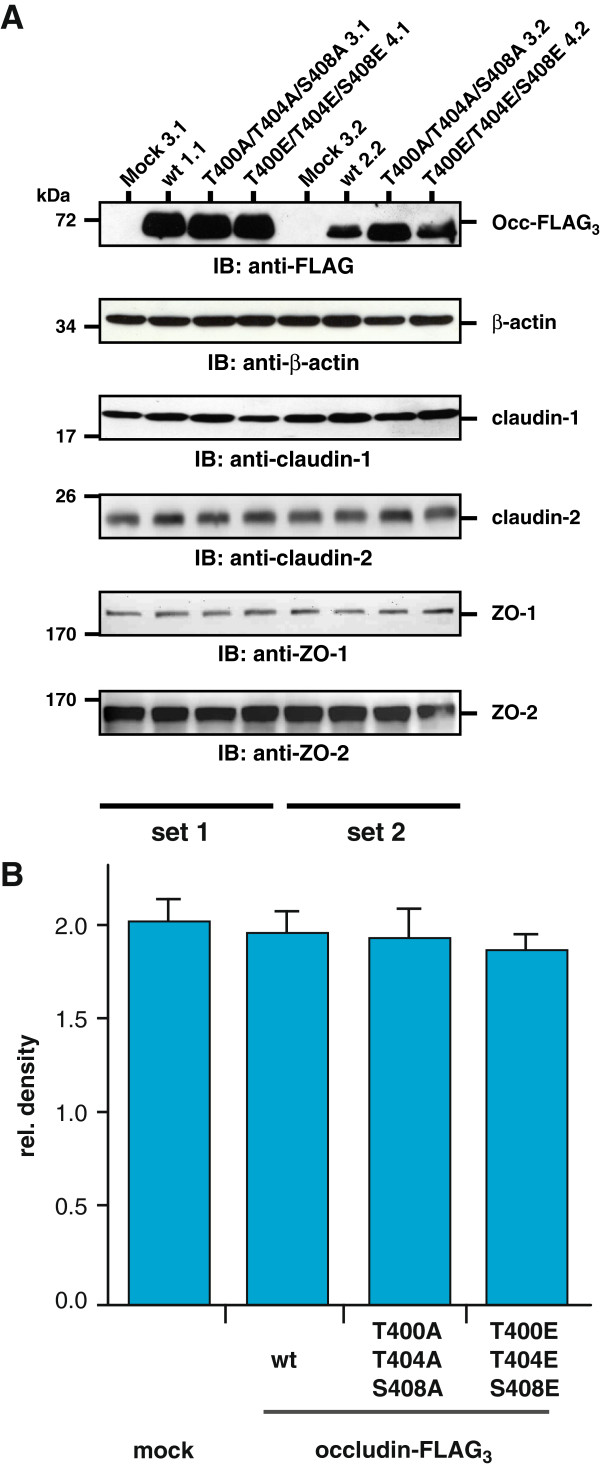
**Expression of TJ proteins in the stably transfected MDCK C11 cells. A**) Claudin-1, claudin-2, ZO-1 and ZO-2 expression is not affected by the stable transfection of the indicated occludin-FLAG_3_ constructs as detected by Western blotting. β-Actin was used as a loading control. Analysis of two different clones for each construct is shown. **B**) Transfection of the indicated occludin constructs does not affect cell proliferation as investigated by a XTT-assay. The graph summarizes the results of 4 experiments including two clones of each construct (mean values +/− SEM).

To exclude that stable transfection with the occludin constructs altered the expression of other TJ components, equal amounts of protein from each of the picked clones were separated by SDS-PAGE and analyzed by Western blotting (Figure 6A). All Occ-FLAG_3_ constructs were expressed at comparable levels within each of the two groups (mock 3.1, wt 1.1, T400A/T404A/S408A 3.1, T400E/T404E/S408E 4.1; mock 3.2, wt 2.2, T400A/T404A/S408A 3.2, T400E/T404E/S408E 4.2), whereas the empty vector control (mock) showed no FLAG-signal. Neither the expression of claudin-1 and -2 nor of ZO-1 and ZO-2 was significantly changed compared to mock-transfected clones. β-Actin was used as a loading control.

Previous studies suggested, that occludin is involved in regulation of cell proliferation [41]. Therefore, the MDCK C11 clones expressing the different occludin-FLAG_3_ constructs were compared in XTT-assays. No significant differences in cell proliferation were detectable (Figure 6B). Taken together stable expression of the phospho-site mutated occludin constructs neither impaired occludin localization or expression of other tight junctional proteins nor affected cell proliferation.

### CK2-dependent phosphorylation affects occludin distribution and dimerization

**Figure 7:**
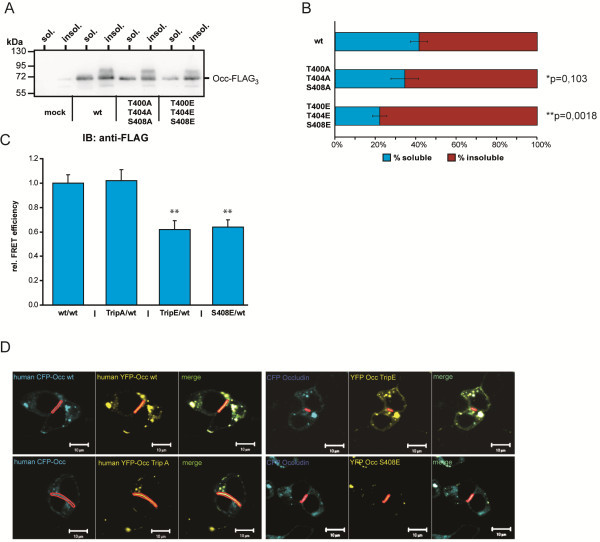
**CK-2-dpendent phosphorylation affects occludin solubility and dimerization.** The distribution of wildtype (wt) occludin (Occ) and of the triple A (TripA) and E (TripE) mutated occludin constructs into TX-100-soluble and -insoluble fractions was analyzed by Western blotting (**A**) and quantified by chemoluminescence imaging (Occ-wt/Occ-TripA *p = 0,103; Occ-wt/Occ-TripE **p = 0,0018; n = 3) (**B**). **C**) Dimerization (cis-interaction) of occludin was analyzed by a fluorescence resonance energy transfer (FRET) assay in cell-cell contacts (see D) between two cotransfected HEK-293 cells. The constructs were N-terminally fused with cyan fluorescent protein (CFP, wt) and yellow fluorescent protein (YFP, mutants). Replacement of serine in human occludin at the position 408 by glutamate reduced the FRET efficiency to wt Occ (S408/wt, n = 40) compared to the wt/wt control (n = 58) similarly as the TripE mutant (TripE/wt, n = 44), whereas the TripA mutant was without any effect (TripA/wt, n **=** 64). **, p < 0.01, Mann–Whitney test. **D**) Fluorescence images of living HEK-293 cells cotransfected with the indicated CFP- and YFP-tagged Occ constructs used for the FRET measurements. Areas of FRET measurement at sites of cell-cell contacts are marked in red. The bar represents 10 μm.

Since phosphorylation does not affect occludin localization to TJs, we next wanted to address, whether its distribution between the TX-100 soluble and insoluble fraction is affected. When wildtype occludin was transfected into MDCK C11 cells about 40% of the FLAG-tagged occludin was found in the TX-100 soluble fraction. In contrast only 22% of the triple E mutated occludin was in the soluble fraction. The triple A mutant did not differ much from wildtype occludin with 35% of the protein in the TX-100 soluble fraction (Figure 7A and B).

Dimerization of occludin is mediated at least in part by its C-terminal domain [42,43] and thus may be affected by CK2-dependent phosphorylation. To address this, FRET analyses were performed in HEK-293 cells. Occludin T400A/T404A/S408A interacts with wildtype occludin similar to homomeric interaction of wildtype molecules. In contrast association of both occludin T400E/T404E/S408E and occludin S408E with wildtype occludin was significantly reduced (Figure 7C,D). In conclusion, these results suggest that transport of occludin to the cell surface and the TJs is not impaired by phosphorylation, however, homodimerization and the interaction with ZO-proteins is affected.

### CK2-dependent phosphorylation of occludin affects TJ-disassembly/assembly in Ca^2+^-switch experiments

**Figure 8:**
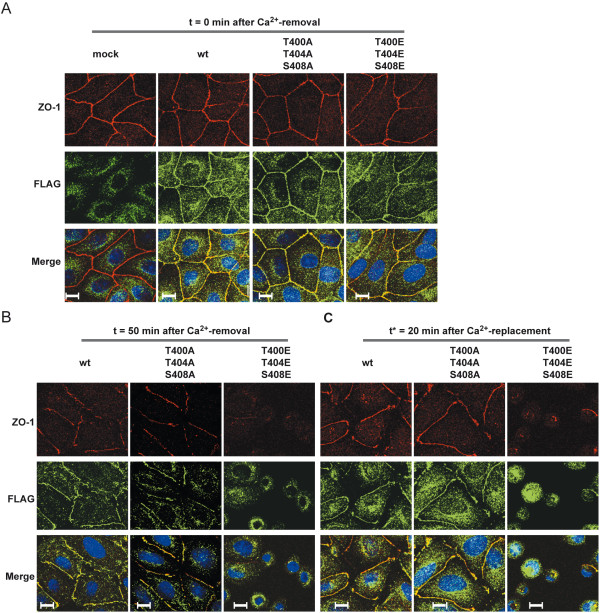
**Phosphorylation of occludin T400/T404/S408 regulates assembly/disassembly of TJs in Ca**^**2+**^**-switch experiments. A**) Confocal images were taken at t = 0 min after removal of Ca^2+^. **B**) After depletion of Ca^2+^ the phospho-mimetic Occ-T400E/T404E/S408E protein is rapidly dissociated from the TJs along with a loss of ZO-1 tight junctional staining. Wildtype occludin and Occ-T400A/T404A/S408A did not differ in the kinetics of disassembly. **C**) After re-addition of Ca^2+^ wildtype occludin and Occ-T400A/T404A/S408A rapidly reassembled into TJs whereas formation of TJs in Occ-T400E/T404E/S408E-transfected MDCK C11 cells was significantly delayed. Confocal images were taken 20 min after addition of Ca^2+^. Bar, 10 μM.

Phosphorylation of occludin was reported to critically regulate TJ assembly and stability [23,39]. In a next step we therefore addressed whether the CK2-phosphorylation site-mutated occludin-FLAG_3_ constructs differ from wildtype occludin-FLAG_3_ during dissociation and reassembly of TJs in Ca^2+^-switch experiments. After removal of Ca^2+^, no change in the kinetics of TJ disassembly was observed between wildtype occludin and Occ-FLAG_3_-T400A/T404A/S408A. In contrast, the phospho-mimetic Occ-FLAG_3_-T400E/T404E/S408E mutant protein dissociates significantly faster from the TJs, as detected by confocal immunofluorescence microscopy (Figure 8A and B). Re-addition of Ca^2+^ had the opposite effect. Both wildtype and the triple A-mutated occludin reappeared at TJs after 20 min whereas the occludin-T400E/T404E/S408E protein was nearly undetectable at intercellular junctions at this time point (Figure 8C). After one hour the triple E occludin mutant protein reappeared at the TJs (not shown). Interestingly, also ZO-1 which was used as a control for tight junctional co-localization was significantly delayed, indicating that not only re-localization of the mutant Occ-FLAG_3_-T400E/T404E/S408E protein was impaired but TJ assembly in general. Similar results were obtained with the other clones tested (not shown). From these observations we conclude, that CK2-dependent phosphorylation of occludin modulates TJ assembly and dynamics.

### OccludinT400E/T404E/S408E increases paracellular resistance

**Figure 9:**
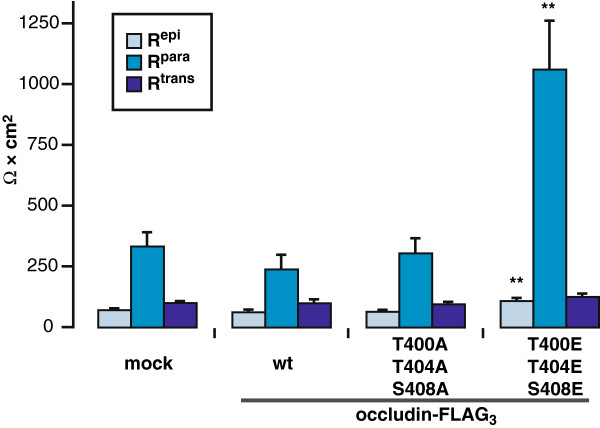
**Increase of paracellular resistance after CK2-dependent phosphorylation of occludin.** Two-path impedance spectroscopy was applied to measure the two components of epithelial resistance (R^epi^), paracellular resistance (R^para^, reflecting the pathway across the tight junctions) and transcellular resistance (R^trans^, reflecting the pathway across the cell membranes). Measurements were done in MDCK C11 cells, which were stably transfected with the indicated occludin-FLAG_3_ constructs. In Occ-FLAG_3_-T400E/T404E/S408E-transfected cells a dramatic increase in R^para^ was detectable compared to wildtype occludin and to Occ-FLAG_3_-T400A/T404A/S408A-transfected cells, while R^trans^ was unchanged. Due to the about fourfold increase of R^para^, the overall epithelial resistance R^epi^ was also increased. The figure shows combined results of 6 independent measurements on two clones of each construct. ** p < 0.01.

To further analyze the physiological consequences of CK2-dependent phosphorylation, the transepithelial resistance of the MDCK C11 clones transfected with the different occludin constructs was measured by two-path impedance spectroscopy, which allows to discriminate between the paracellular resistance (R^para^, reflecting the resistance of the TJs), the transcellular resistance (R^trans^, reflecting the resistance of the apical and basolateral membranes), and the epithelial resistance (R^epi^, which represents the contribution of both resistances). The stable transfection of Occ-FLAG_3_ (R^para^ = 239 ± 59 Ω × cm^2^; R^trans^ = 100 ± 15 Ω × cm^2^; R^epi^ = 63 ± 9 Ω × cm^2^) and Occ-FLAG_3_-T400A/T404A/S408A (R^para^ = 306 ± 60 Ω × cm^2^; R^trans^ = 96 ± 8 Ω × cm^2^; R^epi^ = 65 ± 6 Ω × cm^2^) did not significantly alter the epithelial resistances compared to mock-transfected cells (R^para^ =334 ± 57 Ω × cm^2^; R^trans^ =101 ± 6 Ω × cm^2^; R^epi^ = 71 ± 5 Ω × cm^2^) (n = 12). In contrast, MDCK C11 cells expressing Occ-FLAG_3_-T400E/T404E/S408E showed an about 3-fold increase in R^para^ (1062 ± 199 Ω × cm^2^; **p < 0.005), whereas R^trans^ (126 ± 12 Ω × cm^2^) was again unaltered compared to controls. This results in a 1.5-fold increase in R^epi^ (110 ± 11 Ω × cm^2^; **p = 0.009, n = 12), indicating decreased ion permeability and increased tightness of the epithelium (Figure 9). The depicted data represent mean values obtained from 2 stably transfected clones.

## Discussion

Early after its identification occludin was recognized to be highly phosphorylated at Ser/Thr and Tyr residues. Meanwhile multiple Ser/Thr-kinases including specific PKC isoforms, ERK1/ERK2, CK1 and CK2 (former casein kinase 1 and 2) and Tyr-kinases such as c-Yes and c-Src have been reported to interact with and phosphorylate occludin [16]. In these studies tyrosine-phosphorylation has been associated with disruption of TJs in response to different stimuli and was shown to be low in intact epithelia. In contrast, Ser/Thr-phosphorylation levels are high in resting epithelia. The observed changes in response to removal and readdition of Ca^2+^ in Ca^2+^-switch experiments suggested that Ser/Thr-phosphorylation is a central mechanism regulating recruitment of occludin and its assembly into TJs [44]. Although association of CK2 with occludin has been reported some time ago [30,33], little was known about the potential physiological role of this phosphorylation event. During our analyses a highly sophisticated study by Raleigh et al. addressed this question in very much detail [40]. In the following we specifically discuss our data in the context of the data presented in this paper.

Based on our previous experiments defining Ser408, Thr404 and Thr400 as amino acids targeted by CK2 [31], we here generated FLAG-tagged triple A and triple E phospho-site mutated occludin constructs to mimic unphosphorylated and CK2-phosphorylated occludin. Both mutations did not impair localization of occludin to TJs as shown by confocal immunofluorescence microscopy. Moreover, no changes in cell proliferation and expression levels of other TJ proteins such as claudin-1 and -2 or ZO-1 and -2 were observed. These data are consistent with the observations presented by Raleigh et al., who in addition detected a reduced mobility of occludin after inhibition of CK2 in FRAP experiments. Moreover, occludin-T404A/S408A and occludin-S408A mutant proteins revealed lower mobility fractions compared to wildtype occludin since S408-dephosphorylated occludin is able to interact with ZO-1 which links occludin to selected claudins [40]. In contrast, a GST-occludin cytoplasmic tail S408D fusion protein showed reduced binding to a ZO-1 U5GuK construct compared to the S408A mutated construct [40]. Using FRET analyses we here observed that in contrast to a S408 mono-phosphorylated occludin, triple phosphorylated occludin is able to associate with ZO-1 and in consequence gets integrated into TJs. This is also documented by an enhanced TX-100 insolubility of the phospho-mimetic Occ-T400E/T404E/S408E protein. According to a recent study by Tash et al. defining the primary ZO-1 binding site within residues 468–475 of occludin, CK2-dependent phosphorylation of occludin cannot exert its effects on complex formation directly on this primary interaction site but may act on a postulated secondary site [45]. Interestingly, when we analyzed the binding of ZO-2 protein in pull-down and co-immunoprecipitation experiments, association of ZO-2 was significantly attenuated in the phospho-mimetic Occ-T400E/T404E/S408E-transfected cells, whereas in Occ-T400A/T404A/S408A-transfected cells a minor but not significant increase in ZO-2 binding was detectable compared to wildtype occludin. These findings suggest that ZO-1 and ZO-2 can be differentially regulated. Previous studies with ZO-protein knock-out cell lines or animals have shown that ZO-1 and ZO-2 have overlapping but not fully redundant functions [46-49]. In respect to the dual function of ZO-1 and ZO-2 as Nacos (nuclear and adhesion complexes)-proteins with specific engagement of the proteins in cell adhesion and regulation of gene expression [10,50,51] this suggests that the different effects on ZO-1 and ZO-2 in response to CK2-dependent phosphorylation may represent a mechanism how specific phosphorylation patterns on occludin can modulate cell fate and behavior [52]. This has to be analyzed in more detail in future experiments.

In previous work, the essential ZO-1 binding region was mapped to amino acids 406–488 within occludin which is in close proximity to the CK2 Ser/Thr phosphorylation cluster [43]. Since occludin also dimerizes through a coiled-coil (CC-) domain within this region [26,42,43,53], CK2-dependent phosphorylation may also modulate dimerization of occludin or its interaction with other tight junctional proteins. In experiments using triple A or triple E mutated GST-OccC fusion proteins to pull down FLAG-occludin from transiently transfected HEK-293 cells, we observed that the triple E phospho-mimetic construct bound less FLAG-occludin compared to the GST-OccC T400A/T404A/S408A construct (Additional file 3: Figure S3). However, it has recently been shown that the MARVEL-domain also is involved in dimerization of occludin [54] and thus has to be considered in this respect. Therefore, FRET analyses with full-length occludin constructs which include the MARVEL domain were performed and confirmed that the triple E mutated as well as the S408E occludin is impaired in binding to wildtype occludin.

Since binding of ZO-proteins to occludin is important for regulation of TJ assembly and function [47,55,56], CK2-dependent phosphorylation of occludin may perturb TJ assembly and stability. In this context, we observed an enhanced dissociation of occludin from TJs in response to Ca^2+^-depletion and a significantly delayed reassembly of TJs in response to the switch back to high Ca^2+^-concentrations in MDCK cells transfected with the Occ-T400E/T404E/S408E construct. The kinetics of an Occ-T400A/T404A/S408A construct did not differ from wildtype occludin in this respect. These observations are in contrast to the recently reported effects of PKCη-dependent phosphorylation on TJ integrity [39]: PKCη targets amino acids Thr403 and Thr404 in occludin. Although including an amino acid of the CK2 motif, an exchange of Thr403 and Thr404 to alanine impairs localization of occludin to TJs, whereas the phosphomimetic construct was predominantly detectable at the TJs and, moreover, augmented tight junctional localization of ZO-1. Thus the effects induced by the Occ-T400E/T404E/S408E construct are more comparable to consequences of c-Src-mediated tyrosine phosphorylation of occludin, where a phospho-mimetic Y398D/Y402D construct was impaired in tight junctional localization [23].

To address the physiological consequences of CK2-dependent phosphorylation of occludin on polarized cell layers with fully established tight junctions, the transepithelial resistances of Occ-T400E/T404E/S408E and Occ-T400A/T404A/S408A-transfected MDCK cells were analyzed. Occ-T400E/T404E/S408E-transfected cells showed an enhanced paracellular resistance compared to wildtype and Occ-T400A/T404A/S408A-transfected cells. This can be correlated with binding of the triple E mutated occludin to ZO-1 and its efficient integration into TJs as detected by increased distribution to the TX-100 insoluble fraction. Phosphorylation of occludin T403 and T404 by PKCη also was reported to enhance barrier function [39]. However, in respect to CK2-dependent phosphorylation of occludin our data are in contrast to the reported increase in transepithelial resistance in response to CK2 inhibition or knockdown [40]. In our study a kidney cell line, MDCK C11 was used in which the mutated occludin constructs were expressed on the background of endogenous tight junctional occludin and claudin expression. Different from this, Raleigh et al. used an intestinal cell line, Caco-2, in which they analyzed EGFP-occludin phosphosite-mutated proteins including occludin-T404D/S408D on the background of a knockdown of endogenous occludin. The presence or absence of endogenous wildtype occludin might affect the interactions of the various proteins involved in the formation and function of TJs and might, hence, influence the dynamics and functional features of TJs. Furthermore, the dissimilar tissue origin – intestine versus kidney – and the resulting different endogenous TJ protein expression profile might have affected the observed results. In line with this, it was recently reported that the dynamics of claudins which correlated with their polymerization into TJ strands differed between cell lines [57]. Additionally, it is not clear if these differences depend on the further mutation of T400 or the exchange of threonine and serine residues to glutamate in our construct compared to aspartate. Nevertheless, in line with Raleigh et al. we conclude that posttranslational modifications of occludin differentially affect transport and translocation to the TJs and regulate functional properties when occludin is finally integrated into the TJs. An explanation how this is mechanistically regulated is presented in a model by Raleigh et al. proposing that a change in the mobile fraction of occludin affects its association with ZO-1 and claudin-1 and -2 [40].

During development, occludin phosphorylation seems to be regulated in a stage-specific manner as shown during early mouse and Xenopus development [29,58]. However, the kinase(s) involved in these processes have to be identified. Since CK2 is a constitutively active kinase its cellular functions appear to be regulated by subcellular targeting in response to specific signaling events [35]. Currently, the signals promoting CK2 localization to TJs or targeting it to tight junctional proteins are unknown. It is also not clear whether PKC- and CK2-dependent phosphorylations are mutually exclusive or may occur in parallel. Consensus motifs for PKCs have been characterized to contain basic residues. According to this, pre-phosphorylation by CK2 and subsequent phosphorylation by PKC appears to be very unlikely because CK2 phosphorylation would create a highly negative charge in the neighborhood of the PKC phosphorylation site. However, in this context it has to be noted that the Thr403/Thr404 site in occludin per se does not fit to the typical PKC consensus motif in showing no neighboring basic amino acids. Probably the activity of phosphatases such as PP2A and PP1 [28] play an important role in coordinating phosphorylation of occludin by different kinases. Interestingly, the Thr400-XXX-Thr404-XXX-Ser408 sequence represents a typical glycogen synthase kinase-3β (GSK3β) consensus site, especially when pre-phosphorylated on Ser408. Up to now, we could not detect GSK3β-dependent phosphorylation of occludin neither with nor without pre-phosphorylation with CK2 *in vitro*. Thus the recently reported stabilization of endothelial TJs in response to inhibition of GSK3β [59] may be an indirect effect.

## Conclusions

**Figure 10:**
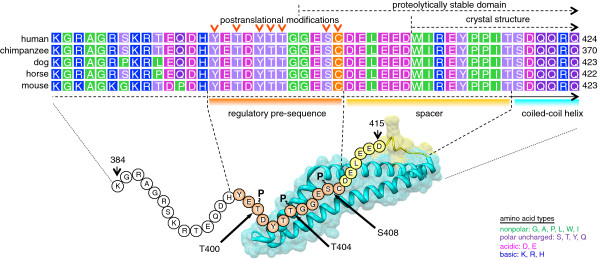
**Scheme of the regulatory sequence in the cytosolic C-terminal coiled-coil (CC)-domain in human occludin depicting the casein kinase 2 (CK2) phosphorylation (~P) sites.** The CC-domain is illustrated by the shaded area calculated from the molecular surface of the crystal structure [42]. The N-terminal part, not resolved by crystallography, is displayed as chain of amino acid circles. Interestingly, various posttranslational modifications (arrowheads) are described in close proximity to the CC-domain within the sequence 398–409 [15,19] including the phosphorylation sites of CK2 (bold arrows), PKCs, Y398 as a c-src phophorylation site and C409 involve in disulfide bond formation. We hence call this segment regulatory pre-sequence of the CC-domain (orange). The link between pre-sequence and the actual CC-helix is thought to function as spacer (yellow). The alignment exhibits that both sequences are highly conserved. The human sequence 407–522 is resistant to proteolytic cleavage [43]. Thus, regulatory pre-sequence, spacer and CC-helix can be considered as functional element.

Taken together, depending on casein kinase 2 and the specifically modified residues in occludin, different effects on TJ assembly and function can be observed most probably depending on the different mobilities of the proteins within the membranes [40] or by a release from raft-like compartments [60]. More important, binding of ZO-proteins is differentially affected. This explains why ZO-1 and ZO-2 have overlapping but not fully redundant functions. In conclusion, the occludin sequence motif at amino acids 400–408 represents a hotspot and regulatory segment for Ser/Thr-kinase phosphorylation [61] including CK2 (Figure 10) which may differentially regulate TJ structure and function.

## Methods

### Cell culture

MDCK C11 and HEK-293 cells were cultured in MEM and DMEM (PAA Laboratories GmbH) respectively, with 10% (v/v) FCS and 100 U/ml penicillin, 100 μg/ml streptomycin under standard cell culture conditions. HEK-293 cells were transiently transfected with calcium phosphate as described previously [62]. Transient transfections of MDCK cells with expression vectors (5 μg of DNA per plate) were performed with Turbofect™ (Fermentas). Stable expressing cells were generated by transfection with 2 μg p3xFLAG-CMV14-Occ expression vectors using FuGENE® HD (Roche). G418-resistant clones were picked and established. Clones transfected with empty vector were generated as control. To confirm stable transfection and analyze the expression of endogenous TJ proteins, cells were grown for 48 h and then washed with ice-cold PBS. Cells were either lysed with lysis buffer (PBS, 0.2% (v/v) Triton X-100, 1 mM NaVO_3_, 10 mM NaF) as described below or membrane fractions were generated by scraping cells from cell culture plates and subsequent homogenization in 0.5 ml lysis buffer containing 20 mM Tris pH 7.5, 5 mM MgCl_2_, 1 mM EDTA and Complete™ protease inhibitor mix (Roche) by passing the cells through a 25 G needle. After centrifugation at 200 × g for 5 min the supernatant was collected and centrifuged for 30 min at 44.000 × g. The resulting pellet was resuspended in 100 μl lysis buffer and total protein concentration was determined using advanced protein assay (Cytoskeleton, Denver; CO, USA). Same amount of protein was separated by SDS-PAGE and blotted with the indicated antibodies.

### Reagents, enzymes and antibodies

Polyclonal antibodies against ZO-1, ZO-2, claudin-1, claudin-2, and occludin were obtained from Zymed (Invitrogen). Mouse monoclonal anti-FLAG-M2 and anti-MBP antibodies were purchased from Sigma, anti-CK2α (clone 1 AD9) and anti-GAPDH antibody was from Chemicon International, anti-HA (6E2) antibody was from Cell Signaling and anti-GST antibody was kindly provided by Jürgen Wienands. HRPO-labeled goat anti-mouse and anti-rabbit antibodies were from Dianova, Alexa Fluor™488 and Alexa-Fluor™594-labeled antibodies were obtained from Molecular Probes (Invitrogen). Enzymes for molecular biology were purchased from Roche, Fermentas or New England Biolabs. Purified CK2 was obtained from New England Biolabs.

### Plasmids

Generation of plasmids to express the cytoplasmic domain of human occludin or fragments thereof (aa263-523, aa263-389 and aa381-523) was reported previously [31]. Full length occludin expression constructs were amplified from occludin cDNA [62] by PCR using the oligonucleotides 5′-GCG GGA TCC ATG TCA TCC AGG CCT CTT G-3′and 5′-CGC GGA TCC CTA TGT TTT CTG TCT ATC ATA GTC-3′ or 5′-CGC GGA TCC GCC GCC ATG TCA TCC AGG CCT CTT GAA-3′ and 5′-GCG GGA TCC TGT TTT CTG TCT ATC ATA GTC TCC-3′ and subcloned into the *BamHI* sites of p3xFLAG-CMV10 and p3xFLAG-CMV14, respectively (Sigma-Aldrich). Briefly, T400, T404 and S408 were mutated to Ala or Glu (single or multiple mutations) with the Change-IT™ Multiple Mutation Site Directed Mutagenesis Kit (USB). The following oligonucleotides were used: 5′-C TAC ACA ACT GGC GGC GAG GCC TGT GAT GAG CTG GAG GAG-3′ (S408A), 5′-C TAT GAG ACA GAC TAC ACA GCT GGC GGC GAG GCC TGT GAT-3′ (T404A/S408A), 5′-GAG CAA GAT CAC TAT GAG GCA GAC TAC ACA GCT GGC GGC-3′ (T400A/T404A), 5′-C TAC ACA ACT GGC GGC GAG GAG TGT GAT GAG CTG GAG GAG-3′ (S408E), 5′-CAG CTC ATC ACA CTC CTC GCC GCC CTC TGT GTA GTC TGT CTC ATA GTG ATC-3′ (T404E/S408E) and 5′-AGA ACA GAG CAA GAT CAC TAT GAG GAA GAC TAC ACA GAG GGC GGC GAG-3′ (T400E/T404E). The sequences of all constructs were verified by resequencing.

### Recombinant protein expression and purification

Fusion proteins of different GST-tagged occludin constructs were expressed in *E. coli* BL21 (DE3). Protein expression was induced at 30°C with 0.5 mM IPTG for 1 h and recombinant proteins were affinity-purified on glutathione (GSH)-agarose (Sigma) as described previously [63].

### In vitro-association assays

To analyze the direct interaction between occludin and CK2 pull-down assays with GST-fusion proteins of different occludin deletion mutants were performed. Purified proteins (2 μg GST-OccC, 0,25 μl CK2) were incubated in pull-down buffer (50 mM Tris/HCl pH 8.0, 50 mM KCl, 0.04% (v/v) Triton X-100, 4 mM MgCl_2_) for 1 h at 4°C under constant agitation. Assays were performed as described previously [64]. Binding of ZO-2 to occludin was investigated using purified GST-OccC fusion protein or corresponding CK2 phosphorylation site mutant proteins, which were incubated with cell extracts obtained from confluent MDCK C11 cell monolayers. Initially, 2 × 10^6^ cells per well were plated and grown for 24 h. Subsequently cells were transfected with 5 μg pGW-HA-cZO-2. After 24 h cells were washed with PBS and incubated with lysis buffer (PBS, 0.2% (v/v) Triton X-100, 1 mM NaVO_3_, 10 mM NaF). After ultrasonification, cellular debris was removed by centrifugation (20.800 × g, 15 min, 4°C). Clarified cell lysates (1 mg total protein) were incubated with 5 μg of each GST-OccC construct or GST protein alone as a control, 30 μl GSH-agarose beads (1:1 slurry) and incubated for 1 h at 4°C under constant agitation. Beads were washed three times with lysis buffer and proteins were eluted by boiling for 5 min in SDS-sample buffer for subsequent analysis by SDS-PAGE and Western blotting. The chemoluminescence signals were analyzed on a Fusion-FX7 system (Vilber Lourmat), the quantification of the signals was performed using ImageJ [65].

### Co-immunoprecipitation assays

For co-immunoprecipitation assays, HEK-293 cells were transiently transfected with 2 μg pRc/CMV-Myc-CK2β, pRc/CMV-HA-CK2α and pFLAG-CMV4-Occludin or corresponding empty vector. Immunoprecipitation assays were performed as described before [62], using the following lysis buffer: 50 mM HEPES, 150 mM NaCl, 300 mM sucrose, 0.05 mM ZnCl_2_, 0.2% (v/v) Triton X-100, pH 6.8. To detect the interaction between occludin and ZO-2, 2 × 10^5^ MDCK C11 cells per well were seeded and incubated for 24 h at 37°C. The cells were transfected with p3xFLAG-CMV14, p3xFLAG-CMV14-occludin, p3xFLAG-CMV14-occludin_T400A/T404A/S408A_ and p3xFLAG-CMV14-occludin_T400E/T404E/S408E_. The cells were lysed 24 h after transfection with 200 μl lysis buffer (PBS, 0.2% (v/v) Triton X-100, 1 mM NaVO_3_, 10 mM NaF) and ultrasonic treatment. Cellular debris was pelleted by centrifugation (20.800 × g, 15 min, 4°C). The supernatant (1.5 mg protein) was incubated with 1 μg anti-ZO-2 antibody for 1 h at 4°C under constant agitation to precipitate endogenous ZO-2. Protein complexes were isolated by incubating the lysates with Protein A-Sepharose (GE-Healthcare) for 30 min at 4°C. Beads were washed with lysis buffer and the pellet was resuspended in 2× SDS-sample buffer, boiled for 5 min and analyzed by SDS-PAGE and Western blotting. Chemoluminescence signals were analyzed on a Fusion-FX7 system and quantification of the signals was performed using ImageJ.

### FRET analyses

For analysis of the cis-interactions between TJ proteins along the cell membrane of one cell, HEK-293 cells were co-transfected with plasmids encoding human occludin wild type as N-terminal fusion protein with cyan fluorescence protein (CFP) and mutants of occludin N-terminally fused with yellow fluorescence protein (YFP), or C-terminally CFP tagged human ZO-1 and the occludin constructs with YFP. Fluorescence resonance energy transfer (FRET) analysis was performed on a Zeiss LSM 510 laser scanning confocal microscope equipped with He/Ne and Ar lasers and a spectral detector. For the microscopy, a Neofluar-Apochromat 100X oil immersion objective NA 1.3 was used. Excitation of CFP was achieved with the Ar laser line at 458 nm (50% power, 3% transmission), of YFP with the Ar laser line at 514 nm (50% Power, 8% transmission). Bleaching of the YFP signal was performed with a YFP excitation laser beam of 100% transmission. FRET was measured after acceptor photobleaching as described previously in living cells [66].

### Immunofluorescence microscopy

For immunofluorescence microscopy 1 × 10^6^ MDCK C11 cells per well were seeded on chamber slides coated with collagen (0.1 μg/μl; Biochrom) and incubated for 24 h at 37°C, 5% CO_2_. Cells were washed with PBS, fixed with methanol (20 min, -20°C) and washed again with PBS. After blocking with 5% (v/v) goat serum (PAA) in PBS for 1 h at RT, cells were stained with anti-FLAG-M2 (5 μg/ml) and anti-ZO-1 (0.5 μg/ml) for 1 h at RT and washed 5 times with PBS. Cells were incubated with Alexa-Fluor488 or Alexa-Fluor594 secondary antibodies and DAPI (0.1 μg/ml) for 30 min at RT, washed 5 times with PBS and cover slides were mounted using ProTaqs Mount Fluor (Bioxyc GmbH&Co. KG). Images were taken on a LSM 510 META confocal laser scanning microscope (Zeiss) with a Plan Apochromat Plan Neofluor objective (63×/1.25 oil) at excitation wavelength 488, 543 or 405 nm. Figures were generated with Adobe Illustrator without further adjustment.

### Cell proliferation

Proliferation of the stable transfected MDCK C11 cells was analyzed using the Cell Proliferation Kit II (Roche) according to the manufacturer’s instructions. In brief, 1 × 10^4^ cells were seeded in 96-well plates in a final volume of 100 μl medium and allowed to grow for 48 h. Subsequently, 50 μl XTT labeling mixture was added, and cells were incubated at 37°C for 4, 6 and 8 h and the absorbance was measured at 450 nm in an ELISA reader.

### Ca^2+^-switch experiments

Stable transfected MDCK C11 clones (1 × 10^6^ cells/35-mm dish) were seeded on cover slides and were allowed to grow for 24 h. Subsequently, cells were rinsed (t = 0) with PBS^−/−^ (without Ca^2+^/Mg^2+^) and incubated with low (LC) calcium medium (SMEM, Invitrogen), containing 10% (v/v) dialyzed FCS, 100 U/ml penicillin, 100 μg/ml streptomycin. At t = 90 min, cells were washed with PBS (t* = 0) and the LC medium was replaced by normal calcium (NC) medium. The cells were fixed at the indicated time points and immuno-stained as described above.

### In vitro-phosphorylation assays

*In vitro* phosphorylation of GST-occludin constructs with CK2 was performed as described before, using radioactive-labeled (P^32^)-γ-ATP [31]. Phosphorylated proteins were separated by SDS-PAGE and radioactivity was measured on a FLA-3000 Fluorescent Image Analyzer (Fujifilm). Data was processed with ImageJ.

### Two-path impedance spectroscopy

Two-path impedance spectroscopy was used to quantify differences in the barrier properties of MDCK C11 cells stably transfected with wildtype FLAG_3_-occludin or the different CK2-site mutated FLAG_3_-occludin constructs as described previously [67]. Cells were seeded on cell culture inserts (Millipore) and allowed to build confluent monolayers. After application of AC (35 μA/cm2, frequency range 1 Hz – 65 kHz), changes in tissue voltage were detected by phase-sensitive amplifiers (402 frequency response analyzer, Beran Instruments, Gilching, Germany; 1286 electrochemical interface; Solartron Schlumberger, Farnborough, United Kingdom). Complex impedance values were calculated and plotted in a Nyquist diagram. R^trans^ and R^para^ were determined from experiments in which impedance spectra and fluxes of fluorescein as a paracellular marker substance were obtained before and after chelating extracellular Ca^2+^ with EGTA. This caused TJs to partly open and to increase fluorescein flux. It was ascertained in separate experiments that changes of fluorescein fluxes are inversely proportional to R^epi^ changes (data not shown).

### Statistics

Data represent mean values ± SEM of at least 3 experiments. Students *t*-test was used to identify significant differences within several experimental groups, with p < 0.05 considered as significant. In case of multiple testing, Bonferroni correction was performed.

## Competing interests

The authors declare no competing interest.

## Authors’ contributions

MJD, JKW, CB, SMK, SM, JC performed experiments. IEB, MF, RT contributed to the conception and design of the experiments and discussion of results, OH designed and coordinated the study and wrote the manuscript. All authors read and fully approved the final version of the manuscript.

## Supplementary Material

Additional file 1: Figure S1 **A**) Co-immunoprecipitation of occludin/ZO-2 complexes from cell lysates of MDCK C11 cells transiently transfected with the indicated occludin-FLAG3 constructs. Endogenous ZO-2 was precipitated with an anti-ZO-2 antibody and association of occludin proteins was analyzed by Western blotting with the anti-FLAG M2 antibody. Rabbit IgG was used as a control for immunoprecipitations. Lysate controls to demonstrate equal transfection and loading are shown below. **B**) Densitometric quantification of 8 experiments as shown in (A). Click here for file

Additional file 2: Figure S2 FLAG-occludin constructs and endogenous ZO-2 were detected by immufluorescence microscopy using an inverted Zeiss Axio Observer.Z.1 ApoTome with a LCI-Plan Neofluor objective (63/1.3 × lm) at excitation wavelenghts of 488, 543 or 405 nm. Bar: 20 μm. Click here for file

Additional file 3: Figure S3 The indicated GST-fusion proteins of the occludin C-terminal cytoplasmic domain were incubated with cell lysates of HEK-293 cells transiently transfected with FLAG-occludin. Associated proteins were pulled down with GSH-agarose beads and detected by Western blotting with anti-FLAG M2 antibody (upper panel). Isolated GST-fusion proteins were detected with an anti-GST antibody (lower panel). GST was used as a control to detect unspecific binding. Click here for file
