# Supplementary material for: CK2-dependent phosphorylation of occludin regulates the interaction with ZO-proteins and tight junction integrity
Source: Cell Commun Signal. 2013 Jun 10;11:40. doi: 10.1186/1478-811X-11-40 (PMC3695765; doi:10.1186/1478-811X-11-40)
Supplement: Additional file 1: Figure S1 — A) Co-immunoprecipitation of occludin/ZO-2 complexes from cell lysates of MDCK C11 cells transiently transfected with the indicated occludin-FLAG3 constructs. Endogenous ZO-2 was precipitated with an anti-ZO-2 antibody and association of occludin proteins was analyzed by Western blotting with the anti-FLAG M2 antibody. Rabbit IgG was used as a control for immunoprecipitations. Lysate controls to demonstrate equal transfection and loading are shown below. B) Densitometric quantification of 8 experiments as shown in (A). [file 1478-811X-11-40-S1.pdf]

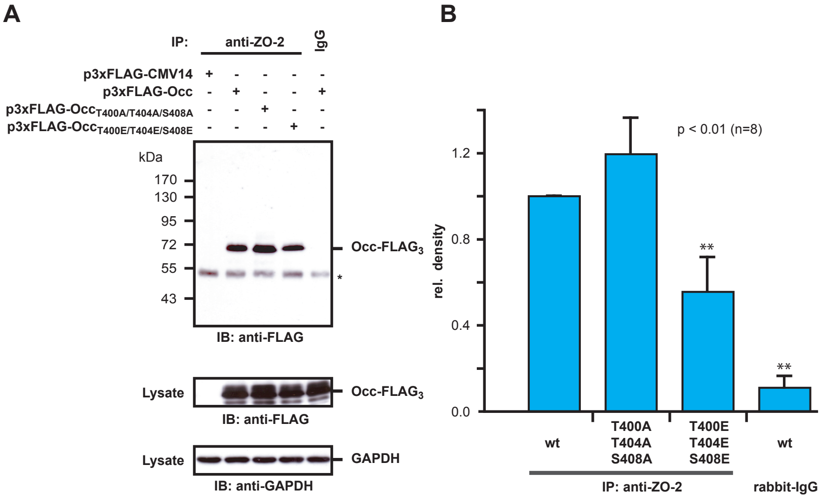

Suppl. Fig. 1:

A) Co-immunoprecipitation of occludin/ZO-2 complexes from cell lysates of MDCK C11 cells transiently transfected with the indicated occludin-FLAG3 constructs. Endogenous ZO-2 was precipitated with an anti-ZO-2 antibody and association of occludin proteins was analyzed by Western blotting with the anti-FLAG M2 antibody. Rabbit IgG was used as a control for immunoprecipitations. Lysate controls to demonstrate equal transfection and loading are shown below. B) Densitometric quantification of 8 experiments as shown in (A).
