# Supplementary figures and images for: CK2-dependent phosphorylation of occludin regulates the interaction with ZO-proteins and tight junction integrity
Source: Cell Commun Signal. 2013 Jun 10;11:40. doi: 10.1186/1478-811X-11-40 (PMC3695765; doi:10.1186/1478-811X-11-40)

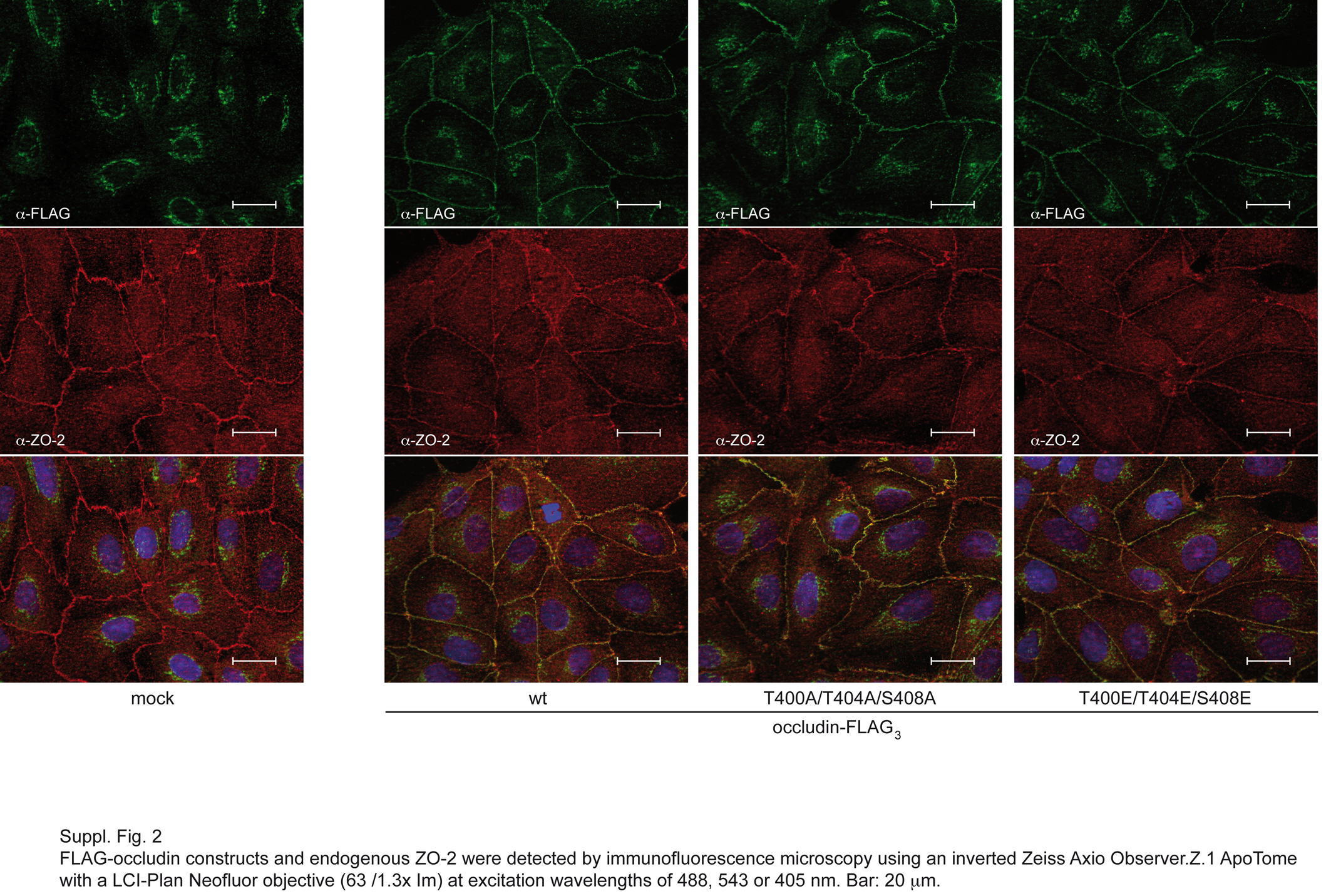

Supplement: Additional file 2: Figure S2 — FLAG-occludin constructs and endogenous ZO-2 were detected by immufluorescence microscopy using an inverted Zeiss Axio Observer.Z.1 ApoTome with a LCI-Plan Neofluor objective (63/1.3 × lm) at excitation wavelenghts of 488, 543 or 405 nm. Bar: 20 μm. [file 1478-811X-11-40-S2.tiff]

A

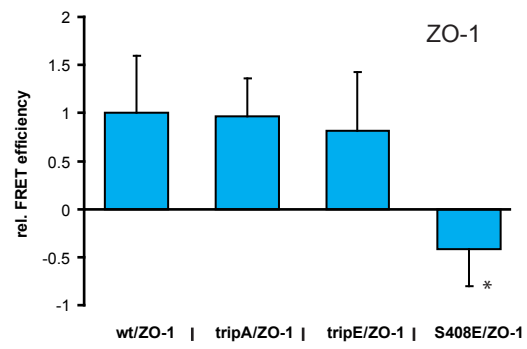

B

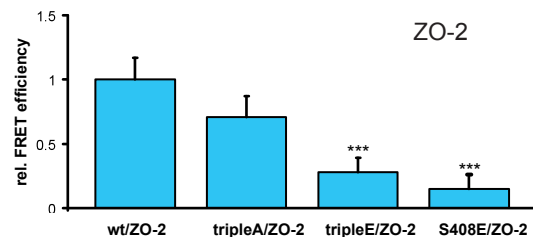

Fig. 3

Supplement: Additional file 3: Figure S3 — The indicated GST-fusion proteins of the occludin C-terminal cytoplasmic domain were incubated with cell lysates of HEK-293 cells transiently transfected with FLAG-occludin. Associated proteins were pulled down with GSH-agarose beads and detected by Western blotting with anti-FLAG M2 antibody (upper panel). Isolated GST-fusion proteins were detected with an anti-GST antibody (lower panel). GST was used as a control to detect unspecific binding. [file 1478-811X-11-40-S3.pdf]
